# Association of lipid management-related knowledge, attitudes, and practices with LDL-C target attainment in patients with coronary heart disease: a multicenter cross-sectional study

**DOI:** 10.1186/s12872-026-06169-x

**Published:** 2026-07-14

**Authors:** Zhihao Lin, Wenjie Yang, Tangjiang Wan, Yucheng Liang, Shitong Shen, Gang Xue, Huan Cao, Jun Zhang, Yafei Li

**Affiliations:** 1 Department of Cardiology, Gusu School, Suzhou Municipal Hospital, the Affiliated Suzhou Hospital of Nanjing Medical University, Nanjing Medical University, Suzhou, 215000 Jiangsu Province China; 2 Department of Cardiology, Anqing Municipal Hospital, Anqing, 246003 Anhui China

**Keywords:** Coronary heart disease, Lipid management, Knowledge-attitude-practice, Low-density lipoprotein cholesterol, Target attainment, Cross-sectional study

## Abstract

**Background:**

Low-density lipoprotein cholesterol (LDL-C) target attainment remains suboptimal in patients with coronary heart disease (CHD). Patients’ knowledge, attitudes, and practices (KAP) regarding lipid management may be associated with LDL-C control; however, evidence from Chinese populations remains limited.

**Methods:**

This multicenter cross-sectional study included 520 patients with CHD from two hospitals in China. Lipid management-related KAP were assessed using a questionnaire developed specifically for this study. LDL-C target attainment was the outcome. Multivariable logistic regression and receiver operating characteristic analysis were performed.

**Results:**

Overall, 22.5% of participants (117/520) achieved the LDL-C target. After adjustment for clinical covariates, attitude (OR = 1.136, 95% CI 1.076–1.203; *P* < 0.001) and practice (OR = 1.213, 95% CI 1.095–1.352; *P* < 0.001) remained significantly associated with LDL-C target attainment, whereas knowledge did not (OR = 1.013, 95% CI 0.878–1.172; *P* = 0.855). Adding KAP variables to the clinical model showed incremental within-sample discriminatory value for LDL-C target attainment, with the area under the curve increasing from 0.772 to 0.817 (*P* = 0.005).

**Conclusions:**

Among Chinese patients with CHD, higher lipid management-related attitude and practice scores were significantly associated with LDL-C target attainment, whereas knowledge scores were not. KAP information showed incremental within-sample discriminatory value beyond the available clinical factors. Given the cross-sectional design, self-developed questionnaire, and lack of detailed lipid-lowering treatment information, these findings should be interpreted as exploratory associations rather than evidence of causal or clinically actionable determinants.

**Supplementary Information:**

The online version contains supplementary material available at https://doi.org/10.1186/s12872-026-06169-x.

## Background

Coronary heart disease (CHD), a clinical manifestation of atherosclerotic cardiovascular disease, remains one of the leading causes of morbidity and mortality worldwide [1]. The Global Burden of Cardiovascular Diseases and Risks 2023 study showed that cardiovascular diseases remain the leading cause of disease burden and death globally, with increasing burden driven by population aging, population growth, and modifiable metabolic risk factors [2]. In China, CHD causes more than 1.5 million deaths each year, and its burden continues to increase with population aging and the growing prevalence of metabolic risk factors [3]. Among the modifiable risk factors for CHD, dyslipidemia is one of the most important. Low-density lipoprotein cholesterol (LDL-C) plays a central role in atherogenesis through arterial lipoprotein retention, while oxidized LDL (Ox-LDL) further promotes endothelial dysfunction and plaque instability, thereby increasing thrombotic risk [4]. As a key driver of atherosclerotic disease, elevated LDL-C is closely associated with the onset and progression of CHD, as well as recurrent cardiovascular events [5]. Accordingly, Chinese and international lipid management guidelines recommend LDL-C as the primary therapeutic target for patients with CHD, with more intensive LDL-C lowering recommended for patients at very high cardiovascular risk [6, 7].

Lipid-lowering therapy (LLT) is essential for achieving LDL-C control and reducing adverse cardiovascular outcomes in patients with CHD. Statins remain the cornerstone of LLT, while newer agents such as proprotein convertase subtilisin/kexin type 9 inhibitors (PCSK9i) have demonstrated additional benefits in reducing cardiovascular events [8]. Nevertheless, despite substantial advances in LLT, suboptimal LDL-C target attainment remains common in routine clinical practice [4, 9]. Previous studies have shown that among patients receiving LLT, the rates of LDL-C target attainment were only 28.48%, 47.60%, 69.12%, and 68.47% in very high-, high-, moderate-, and low-risk populations, respectively, indicating a substantial gap between real-world practice and guideline-recommended goals [10]. This shortfall is particularly pronounced in very high-risk patients and in those requiring intensive lipid-lowering or combination therapy. Persistent failure to achieve LDL-C targets not only increases residual cardiovascular risk, but may also contribute to recurrent acute myocardial infarction and poorer long-term prognosis [11].

The reasons for inadequate LDL-C target attainment are multifactorial and may involve the healthcare system, healthcare professionals, and patients. A survey of physicians from Japan, Germany, Colombia, and the Philippines found cross-country differences in knowledge and clinical practice related to dyslipidemia management, particularly regarding the safety of very low LDL-C levels, statin safety, and lipid management in chronic kidney disease [12]. In addition to provider-level factors, patient-level factors, including disease- and treatment-related knowledge, attitudes, and behavioral practices, may also influence lipid management outcomes. The knowledge–attitude–practice (KAP) framework proposes that knowledge shapes health beliefs and attitudes, which in turn influence the adoption and maintenance of health behaviors. This framework has been widely applied in the management of hypertension, diabetes, and other chronic diseases, and has been shown to be closely associated with treatment adherence and disease control [13, 14]. However, in the context of secondary prevention of CHD, existing KAP research has mainly focused on medication regimens, postoperative self-management, and cardiac rehabilitation [1, 15–17]. Evidence specifically addressing lipid management-related KAP characteristics and their association with LDL-C target attainment remains limited. This gap is particularly evident in Chinese patients with CHD, in whom available multicenter KAP studies have primarily focused on secondary preventive medications or antithrombotic therapy rather than lipid management [1, 3]. Further patient-level research is therefore needed to address this evidence gap.

Against this background, we conducted a multicenter cross-sectional study to systematically assess lipid management-related knowledge, attitudes, and practices among Chinese patients with CHD and to examine the associations between each KAP dimension and LDL-C target attainment. We also investigated whether incorporating KAP information into a model based on conventional clinical variables could provide incremental within-sample discriminatory value for LDL-C target attainment. Our findings may provide patient-level evidence for understanding KAP characteristics associated with LDL-C target attainment in this population.

## Methods

### Ethics approval and consent to participate

This study was approved by the Ethics Committee of Suzhou Municipal Hospital (No. K-2024-191-K03) and the Ethics Committee of Anqing Municipal Hospital (No. Medical Ethics Review [2025] No. 89). Both ethics approvals were obtained before participant recruitment, questionnaire distribution, and data collection. Written or electronic informed consent was obtained from all participants. Electronic questionnaire links were distributed through outpatient clinics, inpatient wards, and a doctor–patient communication platform. For paper-based questionnaires, the informed consent form was presented on the opening page, and questionnaires lacking signed informed consent were excluded. For electronic questionnaires, participants were required to provide consent before accessing the survey. The specific administration mode and recruitment source were not separately recorded in the final study database. Participation was entirely voluntary and did not affect patients’ routine medical care. All participants were assured that the collected data would be kept confidential. Questionnaire responses and clinical data were de-identified before analysis, and personal identifiers were not included in the analytic dataset.

### Study design and participants

A multicenter cross-sectional survey was conducted from October 1, 2025 to February 1, 2026 in the cardiology departments of Suzhou Municipal Hospital and Anqing Municipal Hospital. Patients with confirmed CHD were consecutively recruited during the study period. CHD was defined as coronary angiography-confirmed disease with ≥ 50% stenosis in at least one major coronary artery. The study population may therefore have included patients with different clinical presentations, including acute coronary syndrome, chronic coronary syndrome, prior revascularization, and stable angiographic CHD. All enrolled patients had established CHD and were considered to have established atherosclerotic cardiovascular disease. Therefore, LDL-C < 1.4 mmol/L was used as a stringent lipid-control target in the main analysis according to contemporary lipid management guidelines. Participants were eligible if they met the following criteria: (1) age ≥ 18 years; (2) CHD confirmed by coronary angiography showing ≥ 50% stenosis in at least one major coronary artery; and (3) willingness to participate and provision of informed consent. The exclusion criteria were: (1) severe psychiatric or cognitive impairment, dementia, or other conditions preventing questionnaire completion; and (2) substantial missing questionnaire data or obvious logical inconsistencies, defined as internally contradictory or implausible responses to related items that made the questionnaire unlikely to reflect the participant’s true answers.

### Questionnaire development

The lipid management knowledge–attitudes–practices questionnaire used in this study was developed specifically for the present study and had not been published elsewhere. The English-language version of the questionnaire is provided as Supplementary File 1, and the original Chinese-language questionnaire is provided as Supplementary File 2. Item generation was based on Chinese and international lipid management guidelines and relevant literature [5, 6], and was refined for the context of secondary prevention in patients with CHD. The initial item pool was reviewed and revised by three cardiovascular specialists to ensure the relevance, clarity, and appropriateness of the content.

To assess the validity and reliability of the questionnaire, three rounds of pilot testing were conducted, each involving approximately 30 participants. The questionnaire was iteratively revised after each round. In the third pilot survey, the overall Cronbach’s α coefficient was 0.719, indicating acceptable internal consistency. The final questionnaire comprised four sections. The first section collected basic information, including age, sex, educational level, monthly income, duration of CHD, comorbidities (such as hypertension and diabetes), follow-up status, and exercise habits. The knowledge domain contained 9 items assessing patients’ understanding of LDL-C target levels, the role of lipid-lowering medications, and the influence of lifestyle factors. Items were scored dichotomously (correct = 1; incorrect or uncertain = 0), yielding a total score ranging from 0 to 9. The attitude domain included 10 items rated on a 5-point Likert scale (1 = strongly disagree to 5 = strongly agree) and assessed attitudes toward the importance of lipid management, the necessity of medication, and regular follow-up. Total scores ranged from 10 to 50. For attitude items 7–10, the response options reflected the perceived direction of influence of each factor on appropriate lipid management decisions. “Very positive” and “positive” indicated that the factor was perceived to facilitate or support appropriate lipid management, whereas “negative” and “very negative” indicated that the factor was perceived to hinder appropriate lipid management. Higher scores consistently indicated a more favorable or supportive influence. The practice domain included 5 items rated on a 5-point Likert scale (1 = never to 5 = always) and assessed medication adherence, dietary control, exercise, and regular follow-up behaviors. Total scores ranged from 5 to 25. Higher scores in each domain indicated better knowledge, more positive attitudes, or more favorable practices. In the formal study, the overall Cronbach’s α coefficient of the scale was 0.787, the Kaiser–Meyer–Olkin (KMO) value was 0.831, and Bartlett’s test of sphericity was significant (*P* < 0.001).

### Sample size estimation

The sample size was estimated using the standard formula for cross-sectional studies

$$\:n={\left(\frac{{Z}_{1-\alpha\:/2}}{\delta\:}\right)}^{2}\times\:p(1-p)$$

where *n* is the required sample size, *α* represents the significance level (fixed at 0.05), $\:{Z}_{1-\alpha\:/2}$= 1.96, *δ* is the allowable error (fixed at 0.05), and *p* was assumed to be 0.5 to obtain the maximum required sample size. The minimum required sample size was calculated to be 384. Assuming a 10% invalid questionnaire rate, the target sample size was increased to at least 430 participants. A total of 520 patients were ultimately included, which satisfied the sample size requirement.

### Data collection and variable definitions

General demographic and patient-reported variables, including age, sex, residence, educational level, monthly household income, family history of CHD, history of cardiovascular surgery, regular follow-up, and regular exercise, were collected using the patient-reported questionnaire. Clinical diagnoses and laboratory variables, including angiography-confirmed CHD diagnosis, lipid profiles, diabetes, hypertension, and renal insufficiency, were obtained from the electronic medical record system and laboratory reports. Regular follow-up was defined as undergoing lipid-related follow-up assessment at least three times within the previous 12 months. Regular exercise was defined as engaging in moderate-intensity or higher-intensity physical activity at least three times per week. Detailed lipid-lowering treatment information, including statin intensity and dosage, ezetimibe use, PCSK9 inhibitor use, other non-statin lipid-lowering therapies, treatment duration, baseline LDL-C levels before treatment initiation, and standardized measures of medication adherence, was not systematically collected and was therefore unavailable for analysis.

Fasting venous blood samples were obtained after an overnight fast of at least 8 h. Total cholesterol (TC), triglycerides (TG), and high-density lipoprotein cholesterol (HDL-C) were measured using standard enzymatic methods. LDL-C was calculated using the Friedewald formula or measured directly when TG exceeded 4.5 mmol/L. LDL-C target attainment was defined as an LDL-C level < 1.4 mmol/L (55 mg/dL), which was used as a stringent lipid-control target for this established CHD population according to contemporary Chinese and international lipid management guidelines. Because baseline LDL-C levels before treatment initiation were not systematically collected, percentage LDL-C reduction from baseline could not be assessed.

### Statistical analysis

Statistical analyses were performed using SPSS version 26.0 and R version 4.2.2. Continuous variables were presented as median (interquartile range, IQR) and compared using the Mann–Whitney U test. Categorical variables were presented as frequencies (percentages) and compared using the χ² test or Fisher’s exact test, as appropriate. Participants were classified into attainment and non-attainment groups according to whether the LDL-C target was achieved, and baseline characteristics were compared between the two groups.

The internal consistency of the questionnaire was assessed using Cronbach’s α coefficient. The Kaiser–Meyer–Olkin (KMO) test and Bartlett’s test of sphericity were used to assess the suitability of the data for structural validity analysis. Spearman’s rank correlation analysis was performed to examine associations among the KAP domains. Because LDL-C target attainment was a binary variable, it was coded as 0 for non-attainment and 1 for attainment, and Spearman’s rank-based association analysis was used to explore its monotonic association with KAP scores.

Logistic regression analysis was conducted with LDL-C target attainment as the dependent variable. Univariable logistic regression was first performed for clinical variables. Before multivariable modeling, multicollinearity among candidate independent variables was assessed to avoid instability caused by strong intercorrelations. Two multivariable logistic regression models were then constructed. Covariates in the multivariable models were selected based on clinical relevance, prior evidence, and their potential association with LDL-C target attainment. Model 1 included age group, sex, residence, educational level, monthly household income, family history of CHD, history of cardiovascular surgery, regular exercise, and hypertension. Model 2 further incorporated the total knowledge, attitude, and practice scores in addition to the variables included in Model 1, in order to evaluate the incremental within-sample discriminatory value of KAP information. Results are presented as odds ratios (ORs) with 95% confidence intervals (95% CIs).

Multicollinearity among candidate variables was assessed using variance inflation factors. For categorical variables with more than two levels, adjusted generalized variance inflation factors were calculated. The linearity assumption for continuous KAP scores in the logit was evaluated using spline-based comparisons. The number of LDL-C target attainment events and the events per degree of freedom were calculated to assess the potential risk of model overfitting. Because participants were recruited from two hospitals, an additional sensitivity analysis adjusting for study center was performed.

Receiver operating characteristic (ROC) curves and the area under the curve (AUC) were used to evaluate model discrimination, and the DeLong test was used to compare the AUCs of Model 1 and Model 2. Calibration curves were used to visually assess agreement between predicted probabilities and observed outcomes. All statistical tests were two-sided, and *P* < 0.05 was considered statistically significant.

## Results

A total of 534 questionnaires were distributed. According to the predefined quality-control criteria, 14 questionnaires were excluded as invalid, including 3 completed in less than 60 s, 2 completed in more than 1800 s, 3 with internally inconsistent responses, and 6 with identical responses to all items in the Likert-scale section. Consequently, 520 valid questionnaires were retained in the final analysis. Cronbach’s alpha coefficients were 0.706 for the knowledge domain, 0.794 for the attitude domain, and 0.701 for the practice domain. The overall questionnaire showed acceptable validity, with a Kaiser–Meyer–Olkin value of 0.831 and a significant Bartlett’s test of sphericity (*P* < 0.001).

Baseline characteristics of CHD patients.

Among the 520 participants included in the analysis, 403 did not achieve the LDL-C target and 117 achieved the target (Table 1). Participants in the non-attainment group were older than those in the attainment group [66.00 (59.00, 74.00) vs. 64.00 (56.00, 72.00) years, *P* = 0.028].

**Table 1:** Baseline characteristics according to LDL-C target attainment

Variables	Overall	Not attained	Attained	*P* value
Age (years)	65.00 (58.00, 74.00)	66.00 (59.00, 74.00)	64.00 (56.00, 72.00)	0.028
Age group				0.083
< 55	80 (15.4%)	54 (13.4%)	26 (22.2%)	
55–64	169 (32.5%)	130 (32.3%)	39 (33.3%)	
65–74	167 (32.1%)	133 (33.0%)	34 (29.1%)	
≥ 75	104 (20.0%)	86 (21.3%)	18 (15.4%)	
Height (m)	1.64 (1.55, 1.70)	1.64 (1.55, 1.70)	1.65 (1.57, 1.70)	0.334
Weight (kg)	68.00 (62.00, 77.00)	68.00 (63.00, 77.00)	67.40 (60.00, 76.50)	0.430
BMI (kg/m²)	25.97 (24.06, 27.57)	26.04 (24.32, 27.72)	25.78 (23.53, 27.34)	0.040
BMI group				0.177
< 18.5	9 (1.7%)	5 (1.2%)	4 (3.4%)	
18.5–23.9	111 (21.3%)	82 (20.3%)	29 (24.8%)	
24–27.9	291 (56.0%)	226 (56.1%)	65 (55.6%)	
≥ 28	109 (21.0%)	90 (22.3%)	19 (16.2%)	
Sex				< 0.001
Female	242 (46.5%)	205 (50.9%)	37 (31.6%)	
Male	278 (53.5%)	198 (49.1%)	80 (68.4%)	
Residence				< 0.001
Rural	170 (32.7%)	147 (36.5%)	23 (19.7%)	
Urban	350 (67.3%)	256 (63.5%)	94 (80.3%)	
Education				< 0.001
Junior high school and below	418 (80.4%)	343 (85.1%)	75 (64.1%)	
High school	65 (12.5%)	34 (8.4%)	31 (26.5%)	
College or above	37 (7.1%)	26 (6.5%)	11 (9.4%)	
Family monthly income (CNY)				< 0.001
< 5000	253 (48.7%)	229 (56.8%)	24 (20.5%)	
5000–10,000	214 (41.2%)	142 (35.2%)	72 (61.5%)	
> 10,000	53 (10.2%)	32 (7.9%)	21 (17.9%)	
Family history of CHD	148 (28.5%)	96 (23.8%)	52 (44.4%)	< 0.001
History of cardiovascular surgery	405 (77.9%)	304 (75.4%)	101 (86.3%)	0.018
Diabetes	154 (29.6%)	122 (30.3%)	32 (27.4%)	0.621
Hypertension	475 (91.3%)	382 (94.8%)	93 (79.5%)	< 0.001
Renal insufficiency	47 (9.0%)	37 (9.2%)	10 (8.5%)	0.978
Regular follow-up	311 (59.8%)	245 (60.8%)	66 (56.4%)	0.457
Regular exercise	158 (30.4%)	109 (27.0%)	49 (41.9%)	0.003
Knowledge score	8.00 (5.00, 9.00)	7.00 (4.00, 9.00)	8.00 (6.00, 9.00)	< 0.001
Attitude score	41.00 (35.00, 44.00)	39.00 (35.00, 43.00)	44.00 (40.00, 46.00)	< 0.001
Practice score	20.00 (16.00, 21.00)	19.00 (16.00, 21.00)	21.00 (20.00, 22.00)	< 0.001

KAP scores differed significantly between the two groups. Compared with the non-attainment group, the attainment group had higher knowledge scores [8.00 (6.00, 9.00) vs. 7.00 (4.00, 9.00), *P* < 0.001], attitude scores [44.00 (40.00, 46.00) vs. 39.00 (35.00, 43.00), *P* < 0.001], and practice scores [21.00 (20.00, 22.00) vs. 19.00 (16.00, 21.00), *P* < 0.001].

With respect to sociodemographic and clinical characteristics, the attainment group included a higher proportion of men (68.4% vs. 49.1%, *P* < 0.001), urban residents (80.3% vs. 63.5%, *P* < 0.001), participants with higher educational attainment, and participants with higher monthly household income (both *P* < 0.001). The attainment group also had a higher prevalence of family history of CHD (44.4% vs. 23.8%, *P* < 0.001), prior cardiovascular surgery (86.3% vs. 75.4%, *P* = 0.018), and regular exercise (41.9% vs. 27.0%, *P* = 0.003), whereas hypertension was less common in this group (79.5% vs. 94.8%, *P* < 0.001). No statistically significant between-group differences were observed for height, weight, BMI category, diabetes, renal insufficiency, or regular follow-up (all *P* > 0.05). However, continuous BMI differed significantly between the two groups [25.78 (23.53, 27.34) vs. 26.04 (24.32, 27.72), *P* = 0.040]. Detailed baseline characteristics are presented in Table 1.

### KAP scores across sociodemographic and clinical subgroups

The distribution of KAP scores across sociodemographic and clinical subgroups is presented in Supplementary Table S1. Significant differences in one or more KAP domains were observed according to age group, sex, residence, educational level, monthly household income, family history of CHD, history of cardiovascular surgery, hypertension, regular exercise, and LDL-C target attainment. Overall, participants with urban residence, higher educational attainment, higher household income, a family history of CHD, prior cardiovascular surgery, and regular exercise generally showed more favorable KAP scores. These findings may help describe patient subgroups with different lipid management-related KAP profiles.

### Knowledge dimension of the participants

Responses to the individual knowledge items are summarized in Table 2. This domain mainly covered basic knowledge related to CHD and lipid management, treatment targets, medication strategies, and long-term management. Most items showed relatively high correct response rates; however, performance remained suboptimal for several items. The item with the highest correct response rate was “Cardiovascular disease, mainly coronary heart disease, is the leading cause of death among Chinese residents” (88.5%). In contrast, the lowest correct response rate was observed for “Combination lipid-lowering therapy can improve lipid control” (64.6%), and 32.7% of participants selected “unclear”. In addition, the correct response rate for “Lowering LDL-C is the primary goal of lipid management” was 71.0%, while 29.0% selected “unclear”. Approximately one quarter of participants also selected “unclear” for items related to long-term medication use, treatment adjustment, and long-term lifestyle management.

**Table 2:** Knowledge scores of the participants

	Correct	Incorrect	Unclear
Cardiovascular diseases, mainly coronary heart disease, are the leading cause of death in China.	460(88.5)	6(1.2)	54(10.4)
People with hyperlipidemia usually experience symptoms such as shortness of breath and chest pain.	416(80.0)	3(0.6)	101(19.4)
Lowering low-density lipoprotein cholesterol (LDL-C) can significantly reduce the risk of cardiovascular disease; therefore, reducing LDL-C is the primary target of lipid management.	369(71.0)	0	151(29.0)
Lipid-lowering medications include cholesterol-lowering drugs, triglyceride-lowering drugs, and newer lipid-regulating agents.	386(74.2)	0	134(25.8)
Combination lipid-lowering therapy can improve lipid control.	336(64.6)	14(2.7)	170(32.7)
Long-term use of statins may affect liver function.	380(73.1)	11(2.1)	129(24.8)
During medication use, if symptoms occur, patients should seek medical attention promptly and adjust lipid-lowering therapy according to the doctor’s advice.	382(73.5)	0	138(26.5)
Alcohol consumption is an important factor contributing to elevated blood lipids, and patients with hyperlipidemia should strictly limit alcohol intake.	413(79.4)	0	107(20.6)
Therapeutic lifestyle changes and lipid-lowering drug treatment must be maintained long term to achieve better clinical outcomes.	387(74.4)	0	133(25.6)

### Attitude dimension of the participants

The distribution of responses to the attitude items is shown in Table 3. This domain mainly assessed attitudes toward the importance of lipid management, the safety and effectiveness of lipid-lowering therapy, willingness to adhere to long-term treatment, the importance of regular follow-up, and factors influencing lipid management decisions. Overall, participants demonstrated generally positive attitudes toward lipid management, with “positive” and “very positive” responses accounting for a large proportion of responses across most items. Most participants expressed positive attitudes regarding the importance of lipid management, the effectiveness of lipid-lowering therapy, and the necessity of regular follow-up. However, some participants remained neutral or negative with respect to the safety of lipid-lowering therapy and its potential adverse effects.

**Table 3:** Attitude scores of the participants

	Very positive	Positive	Neutral	Negative	Very negative
Do you think lipid management is important for patients with coronary heart disease?	257(49.4)	145(27.9)	102(19.6)	16(3.1)	0
Do you believe that lipid-lowering therapy is safe?	115(22.1)	283(54.4)	95(18.3)	27(5.2)	0
Do you believe that lipid-lowering therapy is effective for coronary heart disease?	157(30.2)	234(45.0)	119(22.9)	10(2.0)	0
Although lipid-lowering therapy may have adverse effects, would you still be willing to continue taking lipid-lowering therapy in view of its therapeutic benefits?	154(29.6)	218(41.9)	114(21.9)	32(6.2)	2(0.4)
Do you believe that you can follow long-term lipid-lowering therapy as prescribed by your doctor?	163(31.3)	234(45.0)	113(21.7)	10(1.9)	0
Do you think regular examinations and follow-up visits are important during treatment?	224(43.1)	162(31.2)	106(20.4)	28(5.4)	0
Please indicate the extent to which the following factors influence your choice of lipid management:					
a. Family economic status	122(23.5)	250(48.1)	127(24.4)	21(4.0)	0
b. Drug side effects	107(20.6)	62(11.9)	245(47.1)	101(19.4)	5(1.0)
c. Drug effectiveness	241(46.3)	97(18.7)	163(31.3)	19(3.7)	0
d. Doctor’s recommendation	217(41.7)	135(26.0)	143(27.5)	25(4.9)	0

### Practice dimension of the participants

The distribution of responses to the practice items is shown in Table 4. This domain mainly included items related to taking medications as prescribed, forgetting medication, attending regular follow-up visits, adjusting treatment plans according to examination results and physicians’ recommendations, and actively consulting physicians about lipid management. Overall, participants performed relatively well in basic adherence-related behaviors, such as taking medication as prescribed and attending regular follow-up visits. However, they showed comparatively weaker performance in proactive self-management behaviors, including actively seeking medical advice on lipid management and continuously adjusting management strategies according to follow-up results and physicians’ recommendations.

**Table 4:** Practice scores of the participants

	Always	Often	Sometimes	Seldom	Never
I adhere to lipid-lowering therapy as recommended by my doctor and take my medication on time.	174(33.5)	216(41.5)	95(18.3)	35(6.7)	0
I sometimes forget to take my medication.	108(20.8)	201(38.7)	172(33.1)	33(6.3)	6(1.2)
During medication use, I undergo regular examinations and attend follow-up visits.	134(25.8)	199(38.3)	154(29.6)	33(6.3)	0
I adjust the dosage of lipid-lowering medication according to the results of follow-up examinations and my doctor’s advice, based on my own condition.	140(26.9)	164(31.5)	173(33.3)	43(8.3)	0
During medical visits, I actively consult my doctor about knowledge related to lipid management.	154(29.6)	85(16.3)	168(32.3)	111(21.3)	2(0.4)

### Spearman rank-based associations among knowledge, attitude, practice scores and LDL-C target attainment

Spearman rank-based association analyses are presented in Supplementary Table S2. Knowledge, attitude, and practice scores were positively associated with one another (all *P* < 0.01). LDL-C target attainment, coded as a binary variable, showed small-magnitude positive rank-based associations with knowledge, attitude, and practice scores, with coefficients of 0.152, 0.324, and 0.273, respectively.

### Factors associated with LDL-C target attainment

The results of the univariable and multivariable logistic regression analyses are presented in Table 5. Based on clinical relevance and prior evidence, age group, sex, residence, educational level, monthly household income, family history of CHD, prior cardiovascular surgery, regular exercise, and hypertension were included in multivariable Model 1.

**Table 5:** Univariable and multivariable logistic regression analyses for LDL-C target attainment

Variable	Univariable		Multivariable (Model 1)		Multivariable (Model 2)	
	OR (95% CI) *P* value		OR (95% CI) *P* value		OR (95% CI) *P* value	
Age group (years)						
< 55	Ref.					
55–64	0.623 (0.346,1.129)	0.115	1.200(0.564,2.581)	0.637	1.575(0.706,3.578)	0.271
65–74	0.531 (0.291,0.972)	0.039	1.093(0.492,2.450)	0.828	1.084(0.467,2.547)	0.851
≥ 75	0.435 (0.215,0.862)	0.018	0.647(0.268,1.545)	0.328	0.929(0.368,2.336)	0.875
BMI group (kg/m²)						
< 18.5	3.789 (0.869,15.656)	0.063				
18.5–23.9	1.675 (0.879, 3.253)	0.121				
24-27.9	1.362 (0.786, 2.453)	0.285				
≥ 28	Ref.					
Sex						
Female	Ref.					
Male	2.239 (1.457,3.491)	< 0.001	1.059 (0.632,1.774)	0.826	1.004 (0.583–1.728)	0.989
Residence						
Rural	Ref.					
Urban	2.347 (1.447,3.941)	< 0.001	1.205 (0.675–2.192)	0.532	1.123 (0.605–2.121)	0.716
Education						
Junior high school and below	Ref.					
High school	4.170 (2.408,7.219)	< 0.001	2.767 (1.307,5.922)	0.008	3.420 (1.544–7.679)	0.003
College or above	1.935 (0.883,3.996)	0.084	1.293 (0.463–3.515)	0.617	2.108 (0.690–6.310)	0.184
Family monthly income (CNY)						
< 5000	Ref.					
5000–10,000	4.838 (2.952,8.172)	< 0.001	3.424 (1.944,6.160)	< 0.001	1.710 (0.909–3.247)	0.097
> 10,000	6.262 (3.129,12.581)	< 0.001	3.452 (1.544,7.661)	0.002	1.781 (0.742–4.235)	0.192
Family history of CHD						
No	Ref.					
Yes	2.558 (1.661,3.936)	< 0.001	2.147 (1.299,3.546)	0.003	1.877 (1.100–3.197)	0.020
History of cardiovascular surgery						
No	Ref.					
Yes	2.056 (1.187,3.767)	0.014	2.503 (1.333,4.972)	0.006	2.076 (1.041–4.371)	0.045
Regular follow-up						
No	Ref.					
Yes	0.835 (0.551,1.269)	0.395				
Regular exercise						
No	Ref.					
Yes	1.944 (1.264,2.979)	0.002	0.979 (0.544–1.730)	0.942	0.775 (0.411–1.428)	0.420
Hypertension						
No	Ref.					
Yes	0.213 (0.113,0.399)	< 0.001	0.310 (0.148,0.648)	0.002	0.293 (0.132–0.645)	0.002
Diabetes						
No	Ref.					
Yes	0.867 (0.542,1.361)	0.542				
Renal insufficiency						
No	Ref.					
Yes	0.924 (0.423,1.853)	0.833				
Knowledge score	1.218 (1.097,1.36)	< 0.001			1.013 (0.878,1.172)	0.855
Attitude score	1.179 (1.123,1.242)	< 0.001			1.136 (1.076,1.203)	< 0.001
Practice score	1.266 (1.175,1.37)	< 0.001			1.213 (1.095,1.352)	< 0.001

* Knowledge, attitude, and practice scores were entered as continuous variables. Model 1 included age group, sex, residence, educational level, monthly household income, family history of CHD, history of cardiovascular surgery, regular exercise, and hypertension. Model 2 additionally included knowledge, attitude, and practice scores

*OR* odds ratio, *CI* confidence interval, *Ref* reference

In Model 1, educational level, monthly household income, family history of CHD, prior cardiovascular surgery, and hypertension were significantly associated with LDL-C target attainment after adjustment for the available clinical covariates. Compared with participants with junior high school education or below, those with high school education had higher odds of LDL-C target attainment (OR = 2.767, 95% CI 1.307–5.922; *P* = 0.008). Compared with participants with a monthly household income of < 5000 CNY, those with incomes of 5000–10,000 CNY (OR = 3.424, 95% CI 1.944–6.160; *P* < 0.001) and > 10,000 CNY (OR = 3.452, 95% CI 1.544–7.661; *P* = 0.002) had higher odds of LDL-C target attainment. Family history of CHD (OR = 2.147, 95% CI 1.299–3.546; *P* = 0.003) and prior cardiovascular surgery (OR = 2.503, 95% CI 1.333–4.972; *P* = 0.006) were positively associated with LDL-C target attainment, whereas hypertension was negatively associated with LDL-C target attainment (OR = 0.310, 95% CI 0.148–0.648; *P* = 0.002).

After further incorporating knowledge, attitude, and practice scores into Model 1, attitude score (OR = 1.136, 95% CI 1.076–1.203; *P* < 0.001) and practice score (OR = 1.213, 95% CI 1.095–1.352; *P* < 0.001) remained significantly associated with LDL-C target attainment after adjustment for age group and the other available clinical covariates, whereas knowledge score was not significantly associated with target attainment (OR = 1.013, 95% CI 0.878–1.172; *P* = 0.855). High school education, family history of CHD, prior cardiovascular surgery, and hypertension remained statistically significant in Model 2, whereas monthly household income was no longer statistically significant after adding KAP scores.

### Sensitivity analysis additionally adjusted for study center

A total of 117 participants achieved the LDL-C target. The final multivariable model included 16 degrees of freedom, corresponding to approximately 7.3 events per degree of freedom. No serious multicollinearity was observed among candidate variables, with all adjusted generalized variance inflation factors below 2. In spline-based assessments of the linearity assumption for continuous KAP scores in the logit, no obvious nonlinearity was observed for knowledge or practice scores, whereas potential nonlinearity was observed for attitude score and for the overall KAP score specification.

In a sensitivity analysis additionally adjusting for study center, the results were generally consistent with the main analysis. Attitude score (OR = 1.147, 95% CI 1.085–1.216; *P* < 0.001) and practice score (OR = 1.228, 95% CI 1.102–1.376; *P* < 0.001) remained significantly associated with LDL-C target attainment, whereas knowledge score was not significantly associated with target attainment (OR = 0.957, 95% CI 0.822–1.115; *P* = 0.570).

### Incremental discrimination of KAP scores

**Fig. 1:**
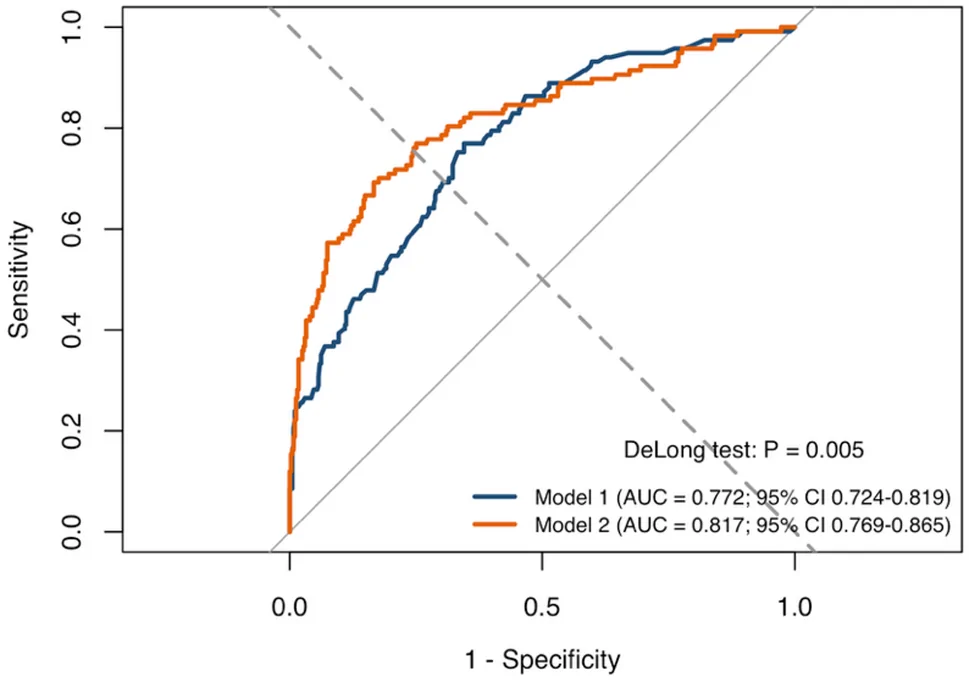
Receiver operating characteristic curves of Model 1 and Model 2 for LDL-C target attainment. Model 1 included clinical variables only, whereas Model 2 additionally incorporated knowledge, attitude, and practice scores. The AUC was 0.772 for Model 1 and 0.817 for Model 2, indicating higher within-sample discrimination after inclusion of KAP scores. The difference in AUCs was assessed using the DeLong test

ROC analysis showed that Model 1 had moderate discriminatory performance for LDL-C target attainment, with an AUC of 0.772 (95% CI 0.715–0.813). After further incorporating knowledge, attitude, and practice scores, Model 2 showed higher within-sample discrimination, with an AUC of 0.817 (95% CI 0.758–0.855). The DeLong test indicated that the difference in AUC between the two models was statistically significant (*P* = 0.005), suggesting incremental within-sample discriminatory value after inclusion of KAP scores (Fig. 1).

**Fig. 2:**
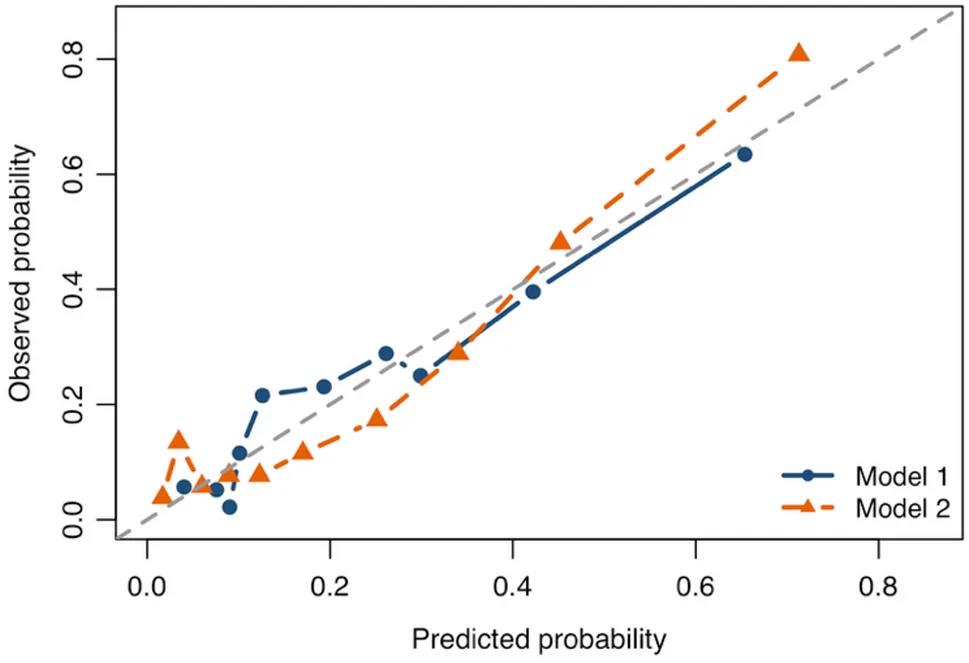
Calibration plots of Model 1 and Model 2 for LDL-C target attainment. The grey dashed diagonal line represents perfect agreement between predicted and observed probabilities. Model 1 included clinical variables only, whereas Model 2 additionally incorporated knowledge, attitude, and practice scores. Calibration was visually assessed by comparing grouped predicted probabilities with observed event rates

Calibration plots were used to visually assess agreement between predicted probabilities and observed outcomes (Fig. 2).

## Discussion

In this multicenter cross-sectional study of patients with CHD from two hospitals in China, we systematically assessed lipid management-related knowledge, attitudes, and practices and examined their associations with LDL-C target attainment. Three main findings emerged. First, the overall LDL-C target attainment rate was only 22.5%, indicating a substantial gap between guideline-recommended lipid goals and real-world lipid management among patients with CHD. Second, after adjustment for relevant demographic and clinical factors, the attitude and practice domains remained significantly associated with LDL-C target attainment, whereas the knowledge domain did not. Third, adding KAP information to conventional clinical variables showed incremental within-sample discriminatory value.

Imaging evidence also supports the biological rationale for intensive LDL-C lowering in patients with coronary atherosclerosis. Intravascular imaging studies have shown that moderate- to high-intensity lipid-lowering therapy is associated with favorable changes in high-risk coronary plaque features, including plaque stabilization and regression [18]. These findings provide visual evidence that sustained LDL-C lowering may be linked to anti-atherosclerotic effects beyond numerical lipid control. Improving patients’ and physicians’ understanding of these imaging-based benefits may help strengthen treatment beliefs and long-term engagement with lipid management.

Lipid management is a central component of secondary prevention in patients with CHD, as it aims to slow the progression of atherosclerotic plaques, stabilize vulnerable plaques, and reduce recurrent cardiovascular events. Despite its clear clinical importance, studies specifically examining lipid management-related KAP remain limited. In the present study, attitudes and practices remained significantly associated with LDL-C target attainment after adjustment for multiple demographic and clinical factors, whereas knowledge did not. This finding suggests that, in lipid management among patients with CHD, disease- or medication-related knowledge alone may not be sufficient to explain variation in LDL-C target attainment. By contrast, patients’ perceived importance of lipid management, treatment beliefs, and sustained behaviors—such as persistent medication use, regular follow-up, dietary control, and lifestyle modification—may show closer associations with LDL-C target attainment in this study population. This interpretation is broadly consistent with the KAP framework, in which knowledge provides an important foundation for behavioral change, but its translation into measurable health outcomes often depends on supportive attitudes and sustained behavioral implementation [19]. Our findings are also consistent with previous evidence. For example, a KAP study in patients with atrial fibrillation reported limited knowledge but relatively positive attitudes and proactive behaviors, suggesting that these three domains do not necessarily change in parallel and that attitudes and behaviors may be more proximal to actual disease management [20]. In the field of lipid management, a prospective study found that LDL-C target attainment was associated not only with medication adherence, but also with patients’ beliefs about their medications [21]. Other studies likewise showed that patients who achieved LDL-C targets generally had better statin adherence than those who did not [22]. Taken together, these findings support our interpretation that, compared with knowledge alone, attitudinal engagement and behavioral implementation may be more closely linked to the achievement of LDL-C control in China.

Importantly, the non-significant association observed for the knowledge domain in the fully adjusted model does not imply that knowledge itself is unimportant. Knowledge may provide an important foundation for lipid management, particularly regarding LDL-C treatment targets and long-term treatment strategies. However, sustained LDL-C target attainment may be more closely associated with stable treatment beliefs and long-term self-management practices [23]. This suggests that knowledge—especially regarding specific therapeutic targets—may still be clinically important. Evidence from the broader health literacy literature in chronic disease further indicates that inadequate health literacy increases the difficulty of self-management. Although health literacy interventions can improve health literacy, self-efficacy, and some health outcomes, they do not necessarily translate into improvement across all long-term clinical outcomes [24, 25]. Thus, knowledge may be a necessary precondition for lipid management, but sustained target attainment may depend more on stable attitudes toward disease risk and treatment benefit, together with the ability to convert knowledge into long-term self-management practices.

The item-level findings further support this interpretation. In the knowledge domain, patients generally demonstrated an acceptable understanding of CHD and dyslipidemia, but important gaps remained in management-relevant knowledge, particularly knowledge of LDL-C treatment targets, combination lipid-lowering therapy, and long-term treatment adjustment. This pattern suggests that the main deficit may lie not simply in a lack of basic knowledge, but in insufficient practical knowledge. Similar findings have been reported in the GOULD registry of dyslipidemia management among patients with ASCVD in the United States, in which only about 10% of patients could identify the LDL-C treatment target of < 70 mg/dL; even among patients receiving PCSK9 inhibitor therapy, this proportion was only about 25% [26]. In the attitude domain, although patients generally recognized the importance of lipid management, a proportion still expressed concerns regarding the safety of lipid-lowering therapy and potential adverse effects. Previous studies have shown that such concerns may negatively affect long-term adherence and LDL-C target attainment [27]. In the practice domain, patients performed relatively well in basic adherence-related behaviors, such as taking medication as prescribed and attending regular follow-up visits, but remained weaker in proactive behaviors, including seeking medical advice, maintaining ongoing engagement in disease management, and sustaining long-term self-management. Taken together with evidence from health literacy and chronic disease self-management research, these findings suggest that LDL-C target attainment may be related not only to knowledge acquisition, but also to stable treatment beliefs and sustained proactive management behaviors [28, 29]. Therefore, the principal weakness in this population may not be a simple lack of knowledge, but rather a disconnect between key treatment knowledge, treatment beliefs, and proactive self-management practices. This may help explain why the attitude and practice domains showed stronger associations with LDL-C target attainment than the knowledge domain.

Beyond the KAP domains, education level, family history of CHD, prior cardiovascular surgery, and hypertension remained associated with LDL-C target attainment after adjustment for the available covariates. Higher educational attainment remained associated with LDL-C target attainment in the fully adjusted model, whereas the association for household income was attenuated after adding KAP scores. These findings suggest that socioeconomic factors may still be relevant to lipid control, although their effects may partly overlap with patient-level KAP characteristics. Patients with higher education and income levels may have greater access to health information, a better understanding of treatment, and more resources to support long-term management. This is broadly consistent with previous evidence showing that lower socioeconomic status is associated with poorer statin adherence and less favorable control of cardiovascular risk factors [30, 31]. We also observed that a family history of CHD was associated with higher odds of LDL-C target attainment. One possible explanation is that patients with a family history may perceive cardiovascular risk more strongly and therefore place greater emphasis on lipid management and preventive treatment. However, evidence regarding the relationship between family history and health behavior remains mixed. Some studies suggest that family history may prompt clinicians to offer more active preventive recommendations and may also heighten patients’ awareness of cardiovascular risk [32], whereas a review found that awareness of a family history of CVD or of one’s own CVD risk does not reliably predict changes in health-related behavior [33]. Accordingly, the association observed in our study may reflect the combined effects of risk perception, healthcare-seeking behavior, and more intensive physician management. Prior cardiovascular surgery was also associated with higher odds of LDL-C target attainment. This may indicate that patients who have undergone cardiovascular procedures receive more intensive follow-up, more frequent clinician contact, and more intensive secondary prevention, which may partly explain the observed association with LDL-C target attainment. Previous studies have reported that patients with recent acute coronary syndrome often fail to achieve LDL-C targets at discharge. Although 98.1% received statins, including 62.3% on statin monotherapy and 35.8% on statin plus ezetimibe, LDL-C target attainment remained suboptimal [34]. These findings suggest that, in patients with high residual risk, further implementation of early intensive lipid-lowering and combination therapy remains necessary. In contrast, hypertension was consistently associated with lower LDL-C target attainment. This is in line with previous studies showing that patients with coexisting hypertension, dyslipidemia, and other cardiovascular risk factors often face greater difficulty in achieving simultaneous control of multiple targets, and LDL-C attainment rates in hypertensive patients are therefore often lower [35, 36]. Potential explanations include multimorbidity, more complex medication regimens, greater behavioral demands, and competing priorities across multiple risk-factor management goals.

We also found that adding KAP domains to a model based on conventional sociodemographic and clinical variables showed incremental within-sample discriminatory value for LDL-C target attainment, with the AUC increasing from 0.772 to 0.817. However, this finding should be interpreted as evidence of incremental within-sample discrimination rather than proof of a clinically generalizable prediction tool. Whether KAP assessment can support targeted educational or behavioral interventions and improve LDL-C control requires validation in prospective studies and intervention trials. Calibration was visually assessed, but the model was not externally validated. Therefore, the potential predictive performance and clinical usefulness of KAP assessment require validation in independent cohorts and prospective studies .These findings suggest that KAP information may provide additional patient-level information beyond traditional variables and may therefore offer a more comprehensive understanding of factors associated with LDL-C control. However, this finding is better interpreted as evidence of the incremental value of KAP for within-sample risk discrimination rather than as proof of a clinically generalizable prediction tool. According to prediction model methodology, apparent performance during model development is often better than performance in independent populations. True generalizability therefore requires external validation, together with further assessment of calibration, overall model performance, and potential clinical utility [37].

The interpretation of these findings should also consider geographical and healthcare-system contexts. This study was conducted in two hospitals in China, where lipid management patterns may be influenced by local healthcare delivery, insurance coverage, medication accessibility, patient education, and follow-up practices. Previous real-world studies from China and the United States have shown that LDL-C target attainment and lipid-lowering therapy use vary substantially across regions and healthcare systems [38, 39].In settings with broader access to intensive lipid-lowering therapies, such as ezetimibe or PCSK9 inhibitors, or with more structured chronic disease management systems, the relationship between patient-level KAP and LDL-C target attainment may differ. Therefore, although our findings provide patient-level evidence from a Chinese CHD population, their generalizability to other geographical regions and healthcare systems should be interpreted with caution.

Several limitations should also be acknowledged. First, KAP data were derived primarily from a self-developed questionnaire. Although the scale demonstrated acceptable internal consistency and structural validity in the formal study, this type of measurement remains susceptible to information bias, recall bias, and social desirability bias [40]. Second, detailed lipid-lowering treatment information was not systematically collected, including baseline LDL-C levels before treatment initiation, statin intensity and dosage, ezetimibe use, PCSK9 inhibitor use, other non-statin lipid-lowering therapies, treatment duration, statin intolerance, physician inertia, cost or access barriers, timing from the index CHD event or revascularization, and standardized medication adherence measures. These factors are important determinants of LDL-C target attainment and may have influenced the observed associations. Therefore, residual confounding related to baseline lipid levels, treatment intensity, medication adherence, and healthcare-related factors cannot be excluded [41]. Third, although all participants had angiography-confirmed CHD and were considered to have established atherosclerotic cardiovascular disease, the study population may have been clinically heterogeneous, including patients with acute coronary syndrome, chronic coronary syndrome, prior revascularization, and stable angiographic CHD. LDL-C target attainment was defined as LDL-C < 1.4 mmol/L (55 mg/dL) in the main analysis, reflecting a stringent lipid-control target for patients with established CHD. However, because baseline LDL-C levels before treatment initiation were unavailable, the percentage reduction in LDL-C from baseline could not be assessed. Therefore, a combined definition incorporating both LDL-C < 1.4 mmol/L and ≥ 50% LDL-C reduction from baseline could not be applied. Alternative LDL-C thresholds, such as < 1.8 mmol/L, were also not evaluated in the present study and should be considered in future studies. Fourth, familial hypercholesterolemia and lipoprotein(a) were not systematically assessed. These lipid-related factors may influence LDL-C control and residual cardiovascular risk and should be incorporated in future studies. Fifth, because this was a cross-sectional study, lipid management-related KAP scores and LDL-C target attainment were assessed at the same time point. Therefore, the temporal sequence between KAP characteristics and LDL-C target attainment could not be established, and causal inferences cannot be drawn. Patients who achieved LDL-C targets may have received more intensive follow-up, better counselling, more frequent post-revascularization care, or more aggressive lipid-lowering therapy, which may also have influenced their attitudes and practices. Therefore, the observed associations should be interpreted as exploratory associations rather than evidence of causal or clinically actionable determinants. Prospective and interventional studies are needed to clarify whether changes in lipid management-related KAP are followed by improvements in LDL-C control. Sixth, questionnaire administration mode and recruitment source were not separately recorded. Therefore, the potential influence of different survey modalities, including paper-based and electronic questionnaires, and different recruitment settings, including outpatient clinics, inpatient wards, and the doctor–patient communication platform, on participant selection could not be evaluated. This may have introduced potential selection bias. In addition, the events per degree of freedom in the fully adjusted model was modest, and spline-based assessments suggested potential nonlinearity for the attitude score. Therefore, logistic regression results based on continuous KAP scores should be interpreted cautiously as average associations across the observed score range. Although the results were generally consistent after additional adjustment for study center, potential model overfitting and residual center-level differences cannot be fully excluded. Finally, the sample was drawn from two institutions in a specific geographic region, and caution is therefore needed when generalizing the findings to other regions or to populations receiving care at different healthcare levels. In addition, although the model was internally evaluated, it has not yet been externally validated in an independent population. Its stability, generalizability, and potential clinical applicability therefore remain to be established.

## Conclusion

In conclusion, LDL-C target attainment remained suboptimal among Chinese patients with CHD. Higher lipid management-related attitude and practice scores were associated with LDL-C target attainment after adjustment for the available clinical covariates, whereas knowledge scores were not. KAP information showed incremental within-sample discriminatory value beyond the available clinical factors. However, given the cross-sectional design and the lack of detailed lipid-lowering treatment information, these findings should be interpreted as exploratory associations rather than evidence of causal or clinically actionable determinants. Future prospective studies incorporating detailed treatment-related information are needed to confirm these findings and clarify the temporal relationship between lipid management-related KAP and LDL-C control.

## Supplementary Information

Supplementary Material 1.

Supplementary Material 2.

Supplementary Material 3.

## Data Availability

The datasets generated and/or analyzed during the current study are not publicly available due to patient privacy and institutional restrictions, but are available from the corresponding author on reasonable request.
