# Supplementary material for: Association of lipid management-related knowledge, attitudes, and practices with LDL-C target attainment in patients with coronary heart disease: a multicenter cross-sectional study
Source: BMC Cardiovasc Disord. 2026 Jul 14;26:892. doi: 10.1186/s12872-026-06169-x (PMC13651333; doi:10.1186/s12872-026-06169-x)
Supplement: Supplementary file 1 [file 12872_2026_6169_MOESM1_ESM.docx]

**Supplementary File 1. English-language version of the lipid management-related knowledge, attitudes, and practices questionnaire used in this study**

**This questionnaire was developed specifically for the present study and has not been published elsewhere. It was used to assess lipid management-related knowledge, attitudes, and practices among patients with coronary heart disease.**

**Part I. Informed consent**

1.I have been informed of the study and agree that the collected data may be used for scientific research.

-Yes

-No

**Part II. Demographic and clinical information**

1. Age: _____ years

2. Height: _____ cm

3. Weight: _____ kg

4. Sex:

- Male

- Female

5. Residence:

- Rural

- Urban

6. Educational level:

- Junior high school or below

- High school

- College or above

7. Average monthly household income:

- <5000 CNY

- 5000–10000 CNY

- >10000 CNY

8. Duration of coronary heart disease: _____ years

9. Comorbidities:

- Hypertension

- Diabetes

- Renal insufficiency

- None of the above

10. Do any of your first-degree relatives have coronary heart disease?

- Yes

- No

11. Do you have a history of cardiovascular surgery or revascularization?

- Yes

- No

12. Clinical data collected from laboratory records
Fasting LDL-C level: _____ mmol/L

13. How many times have you had your blood lipids rechecked during the past 12 months?

- <3 times

- ≥3 times

14. Do you engage in moderate-intensity or higher-intensity physical activity at least three times per week?

- Yes

- No

**Part III. Knowledge domain**

**Instructions:** Please choose one answer for each statement.

**Response options:**
Correct / Incorrect / Unclear

1. Cardiovascular diseases, mainly coronary heart disease, are the leading cause of death in China.
2. People with hyperlipidemia usually experience symptoms such as shortness of breath and chest pain.
3. Lowering low-density lipoprotein cholesterol can significantly reduce the risk of cardiovascular disease; therefore, reducing LDL-C is the primary target of lipid management.
4. Lipid-lowering medications include cholesterol-lowering drugs, triglyceride-lowering drugs, and newer lipid-regulating agents.
5. Combination lipid-lowering therapy can improve lipid control.
6. Long-term use of statins may affect liver function.
7. During medication use, if symptoms occur, patients should seek medical attention promptly and adjust lipid-lowering therapy according to the doctor’s advice.
8. Alcohol consumption is an important factor contributing to elevated blood lipids, and patients with hyperlipidemia should strictly limit alcohol intake.
9. Therapeutic lifestyle changes and lipid-lowering drug treatment must be maintained long term to achieve better clinical outcomes.

**Part IV. Attitude domain**

**Instructions:** Please indicate your attitude toward each item.

**Response options:**
Very positive / Positive / Neutral / Negative / Very negative

1. Do you think lipid management is important for patients with coronary heart disease?
2. Do you believe that lipid-lowering therapy is safe?
3. Do you believe that lipid-lowering therapy is effective for coronary heart disease?
4. Although lipid-lowering therapy may have adverse effects, would you still be willing to continue taking lipid-lowering therapy in view of its therapeutic benefits?
5. Do you believe that you can follow long-term lipid-lowering therapy as prescribed by your doctor?
6. Do you think regular examinations and follow-up visits are important during treatment?

Please indicate the extent to which the following factors influence your choice of lipid management:

7. Family economic status
8. Drug side effects
9. Drug effectiveness
10. Doctor’s recommendation

**Part V. Practice domain**

**Instructions:** Please indicate how often you perform each behavior.

**Response options:**
Always / Often / Sometimes / Seldom / Never

1. I adhere to lipid-lowering therapy as recommended by my doctor and take my medication on time.
2. I sometimes forget to take my medication.
3. During medication use, I undergo regular examinations and attend follow-up visits.
4. I adjust the dosage of lipid-lowering medication according to follow-up examination results and my doctor’s advice, based on my own condition.
5. During medical visits, I actively consult my doctor about knowledge related to lipid management.
