# Supplementary material for: Association of lipid management-related knowledge, attitudes, and practices with LDL-C target attainment in patients with coronary heart disease: a multicenter cross-sectional study
Source: BMC Cardiovasc Disord. 2026 Jul 14;26:892. doi: 10.1186/s12872-026-06169-x (PMC13651333; doi:10.1186/s12872-026-06169-x)
Supplement: Supplementary file 2 [file 12872_2026_6169_MOESM2_ESM.docx]

**补充文件 2. 本研究使用的脂质管理相关知识、态度和行为问卷（中文版）**

本问卷为本研究专门设计，既往未公开发表。该问卷用于评估冠心病患者脂质管理相关的知识、态度和行为。

**第一部分 知情同意**

1. 我已知晓本研究相关内容，并同意所收集资料可用于科学研究。

- 是
- 否

**第二部分 人口学和临床资料**

1. 年龄：_____ 岁

2. 身高：_____ cm

3. 体重：_____ kg

4. 性别：

- 男
- 女

5. 居住地：

- 农村
- 城市

6. 教育程度：

- 初中及以下
- 高中
- 大专及以上

7. 家庭平均月收入：

- <5000 元
- 5000–10000 元
- >10000 元

8. 冠心病病程：_____ 年

9. 合并疾病：

- 高血压
- 糖尿病
- 肾功能不全
- 以上均无

10. 您的一级亲属中是否有人患有冠心病？

- 是
- 否

11. 您是否有心血管手术或血运重建治疗史？

- 是
- 否

12. 从实验室记录中收集的临床资料

- 空腹低密度脂蛋白胆固醇（LDL-C）水平：_____ mmol/L

13. 过去 12 个月内，您复查血脂的次数为：

- <3 次
- ≥3 次

14. 您是否每周至少 3 次进行中等强度或以上的身体活动？

- 是
- 否

**第三部分 知识维度**

**填写说明：请针对每个陈述选择一个答案。**

**选项：正确 / 错误 / 不清楚**

1. 心血管疾病，尤其是冠心病，是我国居民死亡的主要原因之一。

2. 高脂血症患者通常会出现气短、胸痛等症状。

3. 降低低密度脂蛋白胆固醇可显著降低心血管疾病风险，因此降低 LDL-C 是血脂管理的主要目标。

4. 降脂药物包括降低胆固醇的药物、降低甘油三酯的药物以及新型调脂药物。

5. 联合降脂治疗可以改善血脂控制。

6. 长期使用他汀类药物可能影响肝功能。

7. 用药过程中如出现不适症状，应及时就医，并根据医生建议调整降脂治疗方案。

8. 饮酒是导致血脂升高的重要因素，高脂血症患者应严格限制饮酒。

9. 治疗性生活方式改变和降脂药物治疗需要长期坚持，才能获得更好的临床获益。

**第四部分 态度维度**

**填写说明：请根据您的真实想法选择最符合的一项。**

**第 1–6 题选项：非常认同 / 认同 / 中立 / 不认同 / 非常不认同**

1. 您认为脂质管理对冠心病患者重要吗？

2. 您认为降脂治疗是安全的吗？

3. 您认为降脂治疗对冠心病有效吗？

4. 尽管降脂治疗可能存在不良反应，但考虑到其治疗获益，您是否仍愿意继续接受降脂治疗？

5. 您认为自己能够按照医生医嘱长期坚持降脂治疗吗？

6. 您认为治疗期间定期检查和随访重要吗？

**请评价下列因素对您选择脂质管理方案的影响方向和程度：**

**第 7–10 题选项：非常正向 / 正向 / 中立 / 负向 / 非常负向**

7. 家庭经济状况

8. 药物不良反应

9. 药物疗效

10. 医生建议

**第五部分 行为维度**

**填写说明：请根据您实际进行下列行为的频率选择最符合的一项。**

**选项：总是 / 经常 / 有时 / 很少 / 从不**

1. 我会按照医生建议坚持降脂治疗，并按时服药。

2. 我有时会忘记服药。

3. 在用药期间，我会定期进行检查并按时随访。

4. 我会根据复查结果和医生建议，结合自身情况调整降脂药物剂量。

5. 就诊时，我会主动向医生咨询脂质管理相关知识。

**计分说明（研究者使用）**

知识维度：回答正确计 1 分，回答错误或不清楚计 0 分，总分范围为 0–9 分。

态度维度：按 5 级 Likert 评分，第 1–6 题由“非常不认同”至“非常认同”分别计 1–5 分；第 7–10 题由“非常负向”至“非常正向”分别计 1–5 分。总分范围为 10–50 分。

行为维度：按 5 级 Likert 评分。第 1、3、4、5 题由“从不”至“总是”分别计 1–5 分；第 2 题“我有时会忘记服药”为反向计分条目，由“总是”至“从不”分别计 1–5 分。总分范围为 5–25 分。

各维度得分越高，分别表示脂质管理相关知识水平越高、态度越积极、行为越有利。
