# Supplementary material for: Association of lipid management-related knowledge, attitudes, and practices with LDL-C target attainment in patients with coronary heart disease: a multicenter cross-sectional study
Source: BMC Cardiovasc Disord. 2026 Jul 14;26:892. doi: 10.1186/s12872-026-06169-x (PMC13651333; doi:10.1186/s12872-026-06169-x)
Supplement: Supplementary file 3 [file 12872_2026_6169_MOESM3_ESM.docx]

**Supplementary Table 1. Distribution of KAP scores across participant characteristics**

| **Variable** | **n** | **Knowledge score** | | **Attitude score** | | **Practice score** | |
| --- | --- | --- | --- | --- | --- | --- | --- |
|  |  | **Median (P25, P75) P** | | **Median (P25, P75) P** | | **Median (P25, P75) P** | |
| Age group (years) |  |  | 0.048 |  | 0.091 |  | <0.001 |
| <55 | 80 | 8 (6, 9) |  | 43 (36, 45) |  | 21 (20, 21) |  |
| 55–64 | 169 | 8 (5, 9) |  | 41 (35, 44) |  | 20 (16, 21) |  |
| 65–74 | 167 | 7 (4, 9) |  | 43 (35, 44) |  | 20 (16, 21) |  |
| ≥75 | 104 | 8 (5, 9) |  | 39 (35, 44) |  | 19 (15, 21) |  |
| BMI group (kg/m²) |  |  | 0.051 |  | 0.483 |  | 0.740 |
| <18.5 | 9 | 9 (8, 9) |  | 43 (34, 45) |  | 21 (20, 21) |  |
| 18.5–23.9 | 111 | 8 (4, 9) |  | 43 (35, 44) |  | 20 (16, 21) |  |
| 24–27.9 | 291 | 8 (4, 9) |  | 40 (35, 44) |  | 20 (16, 21) |  |
| ≥28 | 109 | 7 (5, 8) |  | 43 (35, 44) |  | 20 (16, 21) |  |
| Sex |  |  | <0.001 |  | 0.038 |  | <0.001 |
| Female | 242 | 7 (4, 8) |  | 39 (35, 44) |  | 16 (15, 21) |  |
| Male | 278 | 8 (5, 9) |  | 43 (36, 44) |  | 21 (16, 22) |  |
| Residence |  |  | 0.007 |  | <0.001 |  | <0.001 |
| Rural | 170 | 6 (4, 9) |  | 36 (34, 44) |  | 16 (15, 21) |  |
| Urban | 350 | 8 (5, 9) |  | 43 (36, 44) |  | 20 (16, 21) |  |
| Education |  |  | <0.001 |  | 0.012 |  | 0.002 |
| Junior high school or below | 418 | 7 (4, 9) |  | 41 (35, 44) |  | 20 (16, 21) |  |
| High school | 65 | 9 (8, 9) |  | 44 (36, 45) |  | 21 (19, 22) |  |
| College or above | 37 | 8 (5, 9) |  | 43 (35, 45) |  | 21 (18, 22) |  |
| Monthly household income |  |  | <0.001 |  | <0.001 |  | <0.001 |
| <5000 | 253 | 6 (4, 9) | <0.001 | 36 (34, 43) | <0.001 | 16 (15, 20) | <0.001 |
| 5000–10000 | 214 | 8 (5, 9) |  | 43 (36, 45) |  | 21 (19, 22) |  |
| >10000 | 53 | 8 (7, 9) |  | 43 (36, 44) |  | 21 (20, 22) |  |
| Family history of CHD |  |  | 0.001 |  | 0.010 |  | 0.003 |
| No | 372 | 7 (4, 9) |  | 40 (35, 44) |  | 20 (16, 21) |  |
| Yes | 148 | 8 (6, 9) |  | 43 (36, 44) |  | 21 (17, 21) |  |
| History of cardiovascular surgery |  |  | <0.001 |  | 0.008 |  | <0.001 |
| No | 115 | 6 (4, 8) |  | 39 (33, 44) |  | 17 (16, 21) |  |
| Yes | 405 | 8 (5, 9) |  | 43 (35, 44) |  | 20 (16, 21) |  |
| Diabetes |  |  | 0.426 |  | 0.670 |  | 0.583 |
| No | 366 | 8 (5, 9) |  | 41 (35, 44) |  | 20 (16, 21) |  |
| Yes | 154 | 8 (4, 9) |  | 41 (35, 44) |  | 20 (16, 21) |  |
| Hypertension |  |  | 0.050 |  | 0.066 |  | 0.043 |
| No | 45 | 8 (6, 9) |  | 43 (35, 45) |  | 20 (20, 21) |  |
| Yes | 475 | 7 (4, 9) |  | 41 (35, 44) |  | 20 (16, 21) |  |
| Renal insufficiency |  |  | 0.382 |  | 0.806 |  | 0.084 |
| No | 473 | 8 (5, 9) |  | 41 (35, 44) |  | 20 (16, 21) |  |
| Yes | 47 | 7 (4, 9) |  | 43 (36, 44) |  | 16 (15, 21) |  |
| Regular follow-up |  |  | 0.809 |  | 0.540 |  | 0.328 |
| <3 times | 209 | 8 (5, 9) |  | 40 (35, 44) |  | 20 (16, 21) |  |
| ≥3 times | 311 | 7 (4, 9) |  | 43 (35, 44) |  | 20 (16, 21) |  |
| Regular exercise |  |  | <0.001 |  | <0.001 |  | <0.001 |
| No | 362 | 7 (4, 9) |  | 40 (35, 44) |  | 20 (16, 21) |  |
| Yes | 158 | 8 (6, 9) |  | 43 (36, 44) |  | 21 (16, 22) |  |
| LDL-C target attainment |  |  | <0.001 |  | <0.001 |  | <0.001 |
| Non-attainment | 403 | 7 (4, 9) |  | 39 (35, 43) |  | 19 (16, 21) |  |
| Attainment | 117 | 8 (6, 9) |  | 44 (40, 46) |  | 21 (20, 22) |  |

*Data are presented as median (P25,P75). P values were calculated using the Mann–Whitney U test for two-group comparisons or the Kruskal–Wallis H test for comparisons among three or more groups.

**Supplementary Table S2. Spearman rank-based associations among knowledge, attitude, practice scores and LDL-C target attainment**

|  | Knowledge | Attitude | Practice | LDL-C target attainment |
| --- | --- | --- | --- | --- |
| Knowledge | 1.000 | 0.207 (P<0.001) | 0.137 (P=0.002) | 0.152 (P<0.001) |
| Attitude | 0.207 (P<0.001) | 1.000 | 0.211 (P<0.001) | 0.324 (P<0.001) |
| Practice | 0.137 (P=0.002) | 0.211 (P<0.001) | 1.000 | 0.273 (P<0.001) |
| LDL-C target attainment | 0.152 (P<0.001) | 0.324 (P<0.001) | 0.273 (P<0.001) | 1.000 |

**Supplementary Table S3. Sensitivity analysis additionally adjusted for study center**

| **Variable** | **OR (95% CI)** | **P value** |
| --- | --- | --- |
| Knowledge score | 0.957 (0.822–1.115) | 0.570 |
| Attitude score | 1.147 (1.085–1.216) | <0.001 |
| Practice score | 1.228 (1.102–1.376) | <0.001 |

*The sensitivity model additionally adjusted for study center, age group, sex, residence, educational level, monthly household income, family history of CHD, history of cardiovascular surgery, regular exercise, and hypertension. Knowledge, attitude, and practice scores were entered as continuous variables. OR, odds ratio; CI, confidence interval.
